# GLP-1 Receptor Agonists in Diabetic Kidney Disease: From Clinical Outcomes to Mechanisms

**DOI:** 10.3389/fphar.2020.00967

**Published:** 2020-06-30

**Authors:** Daiji Kawanami, Yuichi Takashi

**Affiliations:** Department of Endocrinology and Diabetes Mellitus, Fukuoka University School of Medicine, Fukuoka, Japan

**Keywords:** diabetic kidney disease, diabetic nephropathy, GLP-1 receptor agonists, liraglutide, semaglutide, dulaglutide

## Abstract

Diabetic Kidney Disease (DKD) is the leading cause of end stage renal disease (ESRD) worldwide. Glucagon-like peptide 1 receptor agonists (GLP-1RAs) are now widely used in the treatment of patients with type 2 diabetes (T2D). A series of clinical and experimental studies demonstrated that GLP-1RAs have beneficial effects on DKD, independent of their glucose-lowering abilities, which are mediated by natriuresis, anti-inflammatory and anti-oxidative stress properties. Furthermore, GLP-1RAs have been shown to suppress renal fibrosis. Recent clinical trials have demonstrated that GLP-1RAs have beneficial effects on renal outcomes, especially in patients with T2D who are at high risk for CVD. These findings suggest that GLP-1RAs hold great promise in preventing the onset and progression of DKD. However, GLP-1RAs have only been shown to reduce albuminuria, and their ability to reduce progression to ESRD remains to be elucidated. In this review article, we highlight the current understanding of the clinical efficacy and the mechanisms underlying the effects of GLP-1RAs in DKD.

## Introduction

Diabetic kidney disease (DKD) is a global concern because it causes end stage renal disease (ESRD) and affects mortality in diabetic patients. The inhibition of the onset and progression of DKD is an urgent issue, and the development of therapeutic approaches against DKD is required. Furthermore, DKD is an established risk factor for cardiovascular disease (CVD) (Rawshani et al., 2018). Thus, anti-diabetic agents that can attenuate both DKD and CVD have been awaited. A recent meta-analysis demonstrated that SGLT2 inhibitors and glucagon-like 1 receptor agonists (GLP-1RAs) have favorable effects on the cardiorenal outcomes in type 2 diabetes (T2D) (Giugliano et al., 2019; Kristensen et al., 2019). GLP-1RAs improve glucose metabolism by increasing glucose-dependent insulin secretion and suppress the release of glucagon. They have also been shown to have beneficial effects on cardiovascular (CV) risk factors by improving obesity, hypertension, and the lipid profile (Drucker, 2018). Recent CV outcome trials utilizing GLP-1RAs have also investigated renal outcomes. In addition, the elucidation of basic mechanisms underlying the renoprotective effect of GLP-1RAs is progressing. In this review article, we discuss the current understanding of the renoprotective effects of GLP-1RAs from clinical and mechanistic standpoints.

## Therapeutic Targets in Diabetic Kidney Disease

DKD is a risk factor for both ESRD and CVD. Intensive glycemic control is effective for preventing the onset and progression of the early-middle stage of DKD. However, its usefulness for progressed DKD and established CVD remains unclear. Recent clinical trials demonstrated that SGLT2 inhibitors and GLP-1RAs bring beneficial effects on the cardiorenal outcomes of T2D subjects who are at high risk for CVD. T2D patients with severe renal impairment are not eligible for SGLT2 inhibitors, and GLP-1RAs could be an important therapeutic option for these patients. DKD is developed by glucose-dependent and -independent mechanisms, including oxidative stress and inflammation. GLP-1RAs have been shown to have beneficial effects on these factors.

## GLP-1RAs and Renal Outcomes

Accumulating clinical evidence demonstrates that GLP-1 RAs have beneficial effects on renal outcomes. The results of major trials are summarized in **Table 1**.

**Table 1:** Clinical effects of GLP-1RAs on DKD.

Trial	Agents, Follow-up Duration	Subjects	Renal Outcomes	Results
LEADER (n=9,340)	Liraglutide (1.8 mg) vs. placebo, 3.84 years	T2D with high CV risk	New-onset macroalbuminuria, doubling of the serum creatinine level, ESRD, renal death	HR 0.78 (95% CI: 0.67-0.92)
SUSTAIN-6 (n=3,297)	Semaglutide (0.5 mg, 1.0 mg) vs. placebo, 104 weeks	T2D Age >50 with established CVD or CKD stage 3-5 Age >60 with CV risk factors	New or worsening of nephropathy (persistent macroalbuminuria, doubling of the serum creatinine level and CCr < 45 mL/min/1.73 m2, RRT)	HR 0.64 (95% CI: 0.46-0.88)
REWIND (n=9,901)	Dulaglutide (1.5 mg) vs. placebo, 5.4 years	T2D with a previous CV event or CV risk factors	New onset of macroalbuminuria, sustained eGFR decline (≥30%) or RRT	HR 0.85 (95% CI: 0.77-0.93)
AWARD-7 (n=576)	Dulaglutide (0.75 mg, 1.5 mg) vs. placebo, 52 weeks	T2D with moderate to severe CKD (stage 3-4)	Changes in eGFR decline and UACR from baseline	eGFR decline: -1.1 (1.5 mg), -1.5 (0.75 mg), -2.9 (glargine) UACR: no significant differences among groups
ELIXA (n=6068)	Lixisenatide (10-20 μg) vs. placebo, 108 weeks	T2D with recent acute coronary syndrome	Percent change in UACR and eGFR from baseline	eGFR decline: no significant differences among groups UACR: -1.69% (95% CI: -11.69% to 8.30%) in patients with normoalbuminuria, -21.10% (95% CI: -42.25% to 0.04%) in patients with microalbuminuria, -39.18% (95% CI: -68.53% to -9.84%) in patients with macroalbuminuria

These effects are mainly driven by the reduction of albuminuria.

### Liraglutide

In an observational study, 52 weeks of liraglutide treatment was shown to increase the glomerular filtration rate (GFR) (5.4 ml/min/1.73 m^2^) and reduce albuminuria by 50% in overweight T2D patients with stage 3 CKD (De Lucas et al., 2017). A small size randomized controlled trial (RCT) demonstrated that treatment with liraglutide (1.8 mg) for 12 weeks resulted in a reduction of albuminuria by 32% in T2D patients (Von Scholten et al., 2017). In the Satiety and Clinical Adiposity–Liraglutide Evidence (SCALE) Diabetes trial, a total of 846 overweight and obese patients with T2D were randomly assigned to receive 3.0 or 1.8 mg of liraglutide or placebo for 56 weeks. At the end of the study period, the reductions in the albumin-to-creatine ratios (UACR) of the liraglutide (3.0 mg), liraglutide (1.8 mg), and placebo groups were 18.36, 10.79, 2.34%, respectively (Davies et al., 2015). In the LIRA-RENAL trial, 279 T2D subjects with moderate renal impairment [estimated glomerular filtration rate (eGFR) 30–59 ml/min/1.73 m^2^] were randomly assigned to receive liraglutide (1.8 mg) or placebo for 26 weeks. However, liraglutide treatment failed to show significant improvement of the UACR and eGFR trajectory in comparison to placebo (Davies et al., 2016).

The Liraglutide Effect and Action in Diabetes: Evaluation of Cardiovascular Outcome Results (LEADER) study assessed the CV outcome of liraglutide (1.8 mg) in comparison to placebo (Marso et al., 2016b). A total of 9340 participants with a high CV risk, who were ≥50 years of age, with HbA1c ≥7% were randomly assigned to receive placebo or liraglutide (1.8 mg). At baseline, 20.7% of the patients had an eGFR of 30–59 ml/min/1.73 m^2^ and 2.4% had an eGFR of <30 ml/min/1.73 m^2^. Microalbuminuria and macroalbuminuria were present in 26.3 and 10.5% of the participants, respectively. The definitions used for the renal outcomes in the LEADER study were a composite of new-onset persistent macroalbuminuria, persistent doubling of serum creatinine, ESRD, or death due to renal disease (Mann et al., 2017). Over a median follow-up of 3.8 years, liraglutide treatment resulted in less renal outcomes in comparison to placebo [HR 0.78 (95% CI: 0.67–0.92, p=0.03)] (Mann et al., 2017). This observation was largely driven by a reduction in new-onset macroalbuminuria in the liraglutide group in comparison to the placebo group [HR 0.74 (95% CI: 0.60–0.91, p=0.004)]. No significant differences in the doubling of the serum creatinine, initiation of renal replacement therapy (RRT), or renal death were observed between the liraglutide and placebo groups (Mann et al., 2017). In a *post-hoc* analysis of the LEADER trial, liraglutide was shown to reduce the risk of major adverse CV events and all-cause mortality in comparison to placebo in patients with chronic kidney disease (CKD), defined as eGFR < 60 ml/min/1.73 m^2^ and albuminuria (UACR >30 mg/g) (Mann et al., 2018).

### Semaglutide

The SUSTAIN-6 (trial to evaluate cardiovascular and other long-term outcomes with semaglutide in subjects with type 2 diabetes) was a double-blind trial in which T2D patients were randomized to receive either 0.5 or 1.0 mg of once-weekly subcutaneous semaglutide or placebo (Marso et al., 2016a). At baseline, 25.2% of the participants had an eGFR of 30–59 ml/min/1.73 m^2^ and 2.9% had an eGFR of <30 ml/min/1.73 m^2^. The composite renal outcome of this study was new or worsening nephropathy, defined as persistent macroalbuminuria, persistent doubling of the serum creatinine level and creatinine clearance <45 ml/min/1.73 m^2^ or the need for RRT. After a median follow-up of 2 years, the incidence of new or worsening nephropathy in the semaglutide group was lower than that in the placebo group [HR 0.64 (95% CI: 0.46–0.88, p=0.05)]. This result was largely driven by a reduction in new onset macroalbuminuria. No significant changes were observed in ESRD or renal death (Marso et al., 2016a).

The PIONEER-6 trial primarily evaluated the cardiovascular safety of oral semaglutide (14 mg) in comparison to placebo (Husain et al., 2019). A total of 3,183 participants of ≥50 years of age with established CVD or CKD, or ≥60 years of age with CV risk factors were only observed for a median of 15.9 months. At baseline, 26.9% of participants had an eGFR of <60 ml/min/1.73 m^2^. There was no significant reported difference in the eGFR decline from baseline to the end of treatment or in the rate of renal death (Husain et al., 2019). The PIONEER-5 trial showed that semaglutide use in T2D patients with renal impairment (eGFR 30–59 ml/min/1.73 m^2^) was safe and effective (Mosenzon et al., 2019a). Further study is needed to elucidate whether the renoprotective effects of semaglutide are consistent in those individuals.

Currently, the ongoing FLOW is assessing whether or not semaglutide can inhibit worsening of CKD in patients with T2D (https://clinicaltrials.gov/ct2/show/NCT03819153). Renal impairment defined as either an eGFR 50–75 ml/min/1.73 m^2^ and UACR 300–5,000 mg/g or an eGFR 25–50 ml/min/1.73 m^2^ and UACR 100–5,000 mg/g are included in this study. An estimated 3,160 participants are to receive once-weekly subcutaneous semaglutide (starting with 0.25 mg and the dose will be increased to 0.5 mg at 4 weeks and 1 mg at 8 weeks) for up to 5 years. The primary endpoint is the time to the first occurrence of a composite primary outcome event, defined as a persistent eGFR decline (≥50% from baseline), reaching ESRD, renal death, or CV death. This study will elucidate the effects of semaglutide in detail.

### Dulaglutide

The AWARD-7 study assessed the efficacy and safety of dulaglutide in T2D patients with moderate-to-severe CKD (Tuttle et al., 2018). The baseline cystatin C–based eGFR (eGFRcys) and creatinine-based eGFR (eGFRcre) values of the participants were 35.3 ml/min/1.73 m^2^ and 36.0 ml/min/1.73 m^2^, respectively. A total of 577 patients were randomly assigned (1:1:1) to receive once-weekly dulaglutide (1.5 mg), once-weekly dulaglutide (0.75 mg), or daily insulin glargine as basal therapy, all in combination with insulin lispro, for 52 weeks. The renal outcomes were changes in the eGFR and UACR. At 52 weeks, the eGFR decline was −1.1 in the dulaglutide (1.5 mg) group, −1.5 in the dulaglutide (0.75 mg) group, and −2.9 in the glargine group. However, the UACR reduction was not significantly different (Tuttle et al., 2018).

The REWIND study evaluated the cardiovascular safety of dulaglutide (1.5 mg) in comparison to placebo (Gerstein et al., 2019b). In total, 9,901 participants of ≥ 50 years of age with T2D and a history or a high risk of CVD were observed for a median of 5.4 years. The composite renal outcome (the first occurrence of new macroalbuminuria, a sustained decline in eGFR of ≥30% from baseline, or RRT) developed less frequently in participants using dulaglutide in comparison to those using placebo [HR 0.85 (95% CI: 0.77–0.93), p=0.0004]. This result was largely driven by a reduction of albuminuria [HR 0.77 (95% CI: 0.68–0.87), p < 0.001]. The rates of a sustained decline in eGFR [HR 0.89 (95% CI: 0.78–1.01), p=0.066] and the need for RRT showed a downward trend but were not statistically significant [HR 0.75 (95% CI: 0.39–1.44), p=0.39] (Gerstein et al., 2019a).

### Exenatide

The EXSCEL (Exenatide Study of Cardiovascular Event Lowering) trial evaluated the CV safety of exenatide (2 mg weekly). In total, 14,752 participants with T2D (HbA1c 6.5–10.0%) with or without a history of CVD were observed for a median of 3.2 years (Holman et al., 2017). At baseline, 21.6% of the participants had an eGFR of <60 ml/min/1.73 m^2^. Exenatide treatment did not change the eGFR significantly. Macroalbuminuria occurred less (2.2%) in exenatide group compared to placebo group (2.5%) [HR 0.87 (95% CI: 0.70–1.07)]. Neither renal composite 1 (40% eGFR decline, RRT, or renal death) nor composite 2 (composite 1 variables plus macroalbuminuria) was reduced by exenatide in unadjusted analyses; however, renal composite 2 was reduced after adjustment [HR 0.85 (95% CI: 0.74–0.98)] (Bethel et al., 2020). In a *post hoc* analysis of a 52-week randomized trial, exenatide treatment did not alter the renal function (creatinine clearance or eGFR) or the onset/progression of albuminuria in comparison to titrated insulin glargine in overweight T2D patients (Muskiet et al., 2019). Finally, a pooled analysis of RCTs and open-label phase III studies showed that once-weekly exenatide reduced albuminuria 26% (95% CI%: −39.5 to −10%) compared with comparators (Van Der Aart-Van Der Beek et al., 2020). Furthermore, the change in the HbA1c value from baseline did not affect the result, suggesting that once-weekly exenatide reduced albuminuria independent of the glucose-lowering effect (Van Der Aart-Van Der Beek et al., 2020).

### Lixisenatide

In the ELIXA (Evaluation of Lixisenatide in Acute Coronary Syndrome) study, T2D patients with a recent coronary artery event were randomly assigned (1:1) to a lixisenatide (10–20 μg) group or placebo group (Pfeffer et al., 2015). Baseline UACR data were available for 5,978 (99%) of the 6,068 patients who were included in the study. Among them, 19% of the participants had microalbuminuria, and 7% had macroalbuminuria. After 108 weeks, changes in UACR from baseline with lixisenatide were −1.69% [(95% CI: −11.69 to 8.30), p=0.7398] in patients with normoalbuminuria, −21.10% [(95% CI: −42.25 to 0.04), p=0.0502] in patients with microalbuminuria, and −39.18% [(95% CI: −68·53 to −9·84), p=0.0070] in patients with macroalbuminuria. No significant differences in eGFR decline were observed between the treatment groups (Muskiet et al., 2018).

### Albiglutide

Harmony outcomes was a double-blind trial that included a total of 9,463 T2D participants of ≥40 years of age and a history of CVD, who were allocated to an albiglutide (30–50 mg weekly) group or placebo group (Hernandez et al., 2018). After a mean follow-up period of 1.6 years, no significant difference in eGFR decline was observed between the two groups (Hernandez et al., 2018).

## Renoprotection of GLP-1RAs is Largely Dependent on Reduction of Albuminuria

As described above, GLP-1RAs have no clinically important effect on eGFR and hard renal endpoints. In a meta-analysis of 60 studies involving 60,077 T2D patients, GLP-1RAs marginally reduced the UACR in comparison to placebo and other antidiabetic agents, but resulted in no clinically relevant changes in eGFR (Avgerinos et al., 2019). Consistently, a recent meta-analysis that included LEADER (liraglutide), SUSTAIN-6 (semaglutide), REWIND (dulaglutide), EXSCEL (exenatide), ELIXA (lixisenatide), Harmony outcomes (albiglutide), and PIONEER-6 (oral semaglutide), demonstrated that treatment with GLP-1 RAs reduced the composite kidney outcome (development of new-onset macroalbuminuria, decline in eGFR or increase in creatinine, ESRD, or renal death by 17% [HR0.83 (95% CI: 0.78–0.89, p < 0·0001)], mainly driven by a reduction in albuminuria (Kristensen et al., 2019).

## Renoprotective Mechanisms of GLP-1RAs

**Figure 1:**
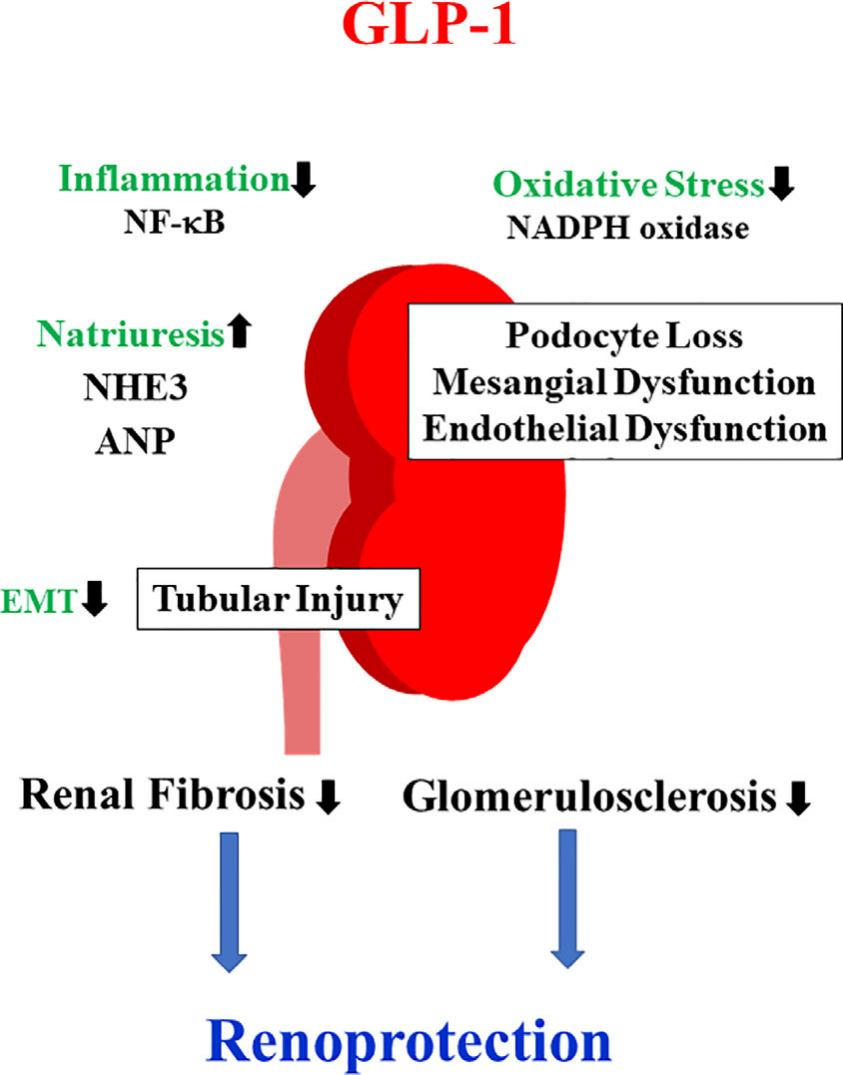
Mechanisms of the renoprotective effects of GLP-1RAs. GLP-1RAs have been shown to activate PKA and increase the production of cyclic adenosine monophosphate (cAMP). As a consequence, nicotinamide adenine dinucleotide phosphate (NADPH) oxidase and NF-κB activity are inhibited, resulting in the attenuation of oxidative stress and inflammation. These favorable effects prevent podocyte loss as well as mesangial and endothelial dysfunction.. GLP-1RAs inactivate NHE3 and promote atrial natriuretic peptide (ANP) secretion, thereby inducing natriuresis. Furthermore, GLP-1RAs inhibit tubular injury and subsequent tubulointerstitial fibrosis.

Experimental studies to elucidate the beneficial effects on DKD have been extensively reported. The inhibition of oxidative stress, inflammation, fibrosis, and induction of natriuresis have been mainly implicated as mechanisms underlying the attenuation of DKD by GLP-1RAs (**Figure 1**).

### GLP-1 Receptors in the Kidney

The distribution of GLP-1R in the kidney is controversial. GLP-1R has been shown to be expressed in the renal cortex as well as the proximal tubules (Schlatter et al., 2007; Carraro-Lacroix et al., 2009). However, several investigations reported a lack of GLP-1R in tubules (Pyke et al., 2014; Lee et al., 2015; Ronn et al., 2017). Studies using monoclonal antibodies against GLP-1R revealed that it is mainly present in the vasculature of the kidney (Pyke et al., 2014; Jensen et al., 2015; Ronn et al., 2017). To date, the presence of GLP-1R in the renal vasculature has been confirmed but not in the tubules (Hviid and Sorensen, 2020).

### Oxidative Stress/Inflammation

GLP-1RAs have been shown to prevent renal oxidative stress by inhibiting nicotinamide adenine dinucleotide phosphate (NADPH) oxidase through the activation of PKA and the production of cyclic adenosine monophosphate (cAMP). Recombinant human GLP-1 inhibits protein kinase C (PKC)-β, but increases protein kinase A (PKA), which reduces oxidative stress in both glomeruli and tubules (Yin et al., 2019). Consistent with this observation, the combination of olmesartan and exenatide has been shown to attenuate the renal NADPH oxidase 4 (Nox4) expression in insulin-resistant Otsuka Long-Evans Tokushima Fatty (OLETF) rats (Rodriguez et al., 2020). Hendarto et. al. revealed that liraglutide attenuates oxidative stress and albuminuria in streptozotocin (STZ)-diabetic rats *via* the PKA-mediated inhibition of renal NADPH oxidases (Hendarto et al., 2012). Liljedahl et al. performed label-free shotgun mass spectrometry (MS) and demonstrated that liraglutide increased the abundance of structurally involved proteins as well as proteins involved in oxidative stress responses in the kidney of STZ-induced diabetic mice (Liljedahl et al., 2019). Moreover, it is reported that exendin-4 inhibits mesangial fibrotic responses (Li et al., 2012; Xu et al., 2014). Exendin-4 has been shown to reduce advanced glycation end product (AGE)-induced interleukin (IL)-6 and TNF-α production, the expression of receptor for AGE (RAGE), and cell death in mesangial cells (Chang et al., 2017). The transcription factor nuclear factor erythroid 2-related factor 2 (Nrf2) and Kelch-like ECH-associated protein1 (Keap1) signaling pathways play an important role in preventing oxidative stress (Wang et al., 2014). Nrf2 activator bardoxolone methyl is known to have renoprotective effects (Ito et al., 2020). Interestingly, exendin-4 has been shown to activate the Nrf2 signaling pathway in vascular smooth muscle cells (Zhou et al., 2016) and retinal pigment epithelial cells (Cui et al., 2019). A further study to investigate whether a similar mechanism exists in kidney under diabetic conditions would be intriguing.

NF-κB plays a central role in the inflammatory pathway in the development of DKD (Kawanami et al., 2016). The hyperglycemia-induced downregulation of GLP-1R is involved in NF-κB activation and the subsequent inflammatory response in mesangial cells (Kang et al., 2019). Liraglutide has been shown to increase renal endothelial nitric oxide synthase (eNOS) levels by downregulating NF-κB in STZ-induced diabetic rats (Zhou et al., 2014). Furthermore, liraglutide inhibits the expression levels of TNF-α-mediated NF-κB activation in podocytes (Ye et al., 2019). Kodera et. al. reported that exendin-4 attenuates albuminuria and glomerulosclerosis independent of the glucose-lowering effect in STZ-induced diabetic rats by inhibiting oxidative stress and NF-κB activation. From a mechanistic standpoint, these observations are mediated by GLP-1R in monocytes/macrophages and glomerular endothelial cells (Kodera et al., 2011). Ye et al. showed that liraglutide attenuated the morphology and structure damage of podocytes in obesity-related glomerulopathy model mice (Ye et al., 2019). Mechanistically, they found that liraglutide inhibited the renal TNF-α expression and NF-κB as well as the MAPK pathway activation in these mice (Ye et al., 2019). Similarly, liraglutide has been shown to reduce renal lipid accumulation and improve the mitochondrial function by activating the Sirt1/AMPK/PGC1α pathways in an obesity-induced rat CKD model (Wang et al., 2018). In addition to the MAPK pathway, the JAK/STAT signaling pathway is also involved in liraglutide-induced renoprotection. Zitman-Gal et al. revealed that liraglutide attenuated the phosphorylation of JAK2 and STAT3 in AGE-stimulated endothelial cells and the kidney of db/db mice (Zitman-Gal et al., 2019). Furthermore, it has been reported that the administration of exenatide attenuates the renal inflammation index, including reducing the TNF-α, IL-6, hsCRP, and CCL5 levels, in STZ-induced diabetic rats by increasing the superoxide dismutase and decreasing malondialdehyde levels (Wang et al., 2019). Taken together, the prevention of oxidative stress and inflammation is a key mechanism for the renoprotective effects of GLP-1RAs.

### Natriuresis

Acute infusion of GLP-1 has been shown to stimulate diuresis and natriuresis in both experimental (Crajoinas et al., 2011; Jensen et al., 2015) and human studies (Gutzwiller et al., 2004; Skov et al., 2013; Muskiet et al., 2016). These observations seem to be associated with the inhibition of Na^+^/H^+^ exchanger 3 (NHE3) in the proximal tubules. NHE3 plays an important role in reabsorbing filtered Na^+^ in the proximal tubules (Schultheis et al., 1998). Therefore, inactivation of NHE3 can result in natriuresis. GLP-1 RAs have been shown to induce phosphorylation and inactivation of NHE3 (Carraro-Lacroix et al., 2009; Crajoinas et al., 2011; Farah et al., 2016; Muskiet et al., 2017). The long-term administration of lixisenatide has been shown to decrease NHE3 activity in overweight T2D patients. In this study, 35 participants were randomly allocated to a lixisenatide (20 mg) group or once-daily insulin glulisine treatment group. After 8 weeks of follow-up, the administration of lixisenatide increased the phosphorylation of NHE3, which reduced its activity in urinary extracellular vesicles in comparison to once-daily insulin glulisine treatment (Tonneijck et al., 2019). However, it remains unclear whether these natriuretic responses are direct effects of GLP-1RA because a lack of GLP-1R in the proximal tubules has been reported (Pyke et al., 2014; Lee et al., 2015; Ronn et al., 2017). Furthermore, cardiomyocyte GLP-1R plays an important role in natriuresis. It has been shown that liraglutide promotes natriuresis by atrial natriuretic peptide (ANP) secretion from cardiomyocytes in an Epac2-depdenent manner (Kim et al., 2013).

### Fibrosis

GLP-1RAs have been shown to attenuate renal fibrosis. For instance, exendin-4 has been shown to ameliorate the high glucose-induced fibronectin (FN) and type I collagen (Col1) expression in tubular epithelial cells by inhibiting the secretion of miR-192, an microRNA (miRNA) that is regulated by p53 and plays a role in renal fibrosis (Jia et al., 2018). Consistent with this observation, liraglutide has been shown to attenuate unilateral ureteral obstruction (UUO)-induced tubulointerstitial fibrosis by suppressing TGF-β and its downstream signaling pathways, including Smad3 and ERK1/2 (Li et al., 2018). These protective effects of GLP-1RAs for renal fibrosis are also mediated by attenuating the epithelial-to-mesenchymal transition (EMT) of tubular cells (Li et al., 2018; Yin et al., 2018).

### The Endothelial Function

DKD is associated with endothelial dysfunction (Chen et al., 2020). Endothelial GLP-1R has been shown to be involved in endothelial dysfunction in a mouse angiotensin II-induced hypertension model (Helmstadter et al., 2020). Liraglutide was found to increase eNOS phosphorylation and nitric oxide (NO) production *via* AMPK-dependent pathways in endothelial cells (Li et al., 2016; Honda et al., 2018; Han et al., 2019). Lixisenatide has also been shown to prevent the free fatty acid-induced reduction of eNOS phosphorylation in endothelial cells (Zhao et al., 2019). Sukumaran et al. showed that liraglutide improves the renal endothelial dysfunction in obese Zucker rats on a high-salt diet by increasing the renal eNOS expression (Sukumaran et al., 2019). They also found that liraglutide increases the NO-mediated vasodilation of small intrarenal arteries using X-ray microangiography (Sukumaran et al., 2019). Furthermore, exendin-4 has been shown to attenuate lipotoxicity-induced glomerular endothelial cell dysfunction in diabetic ApoE-deficient mice by increasing the ABC transporter A1-mediated cholesterol efflux (Yin et al., 2016). Taken together, these findings highlight the improvement of the glomerular endothelial dysfunction as an important renoprotective effect of GLP-1RA.

### Cleavage Products of GLP-1

Moellmann et al. reported that cleavage products derived from GLP-1 reduced tubulointerstitial renal damage, lowered the expression of tubular injury markers, and attenuated the renal accumulation of macrophages and T cells (Moellmann et al., 2018). These findings suggest that GLP-1R-independent renoprotective effects are mediated by GLP-1 cleavage products. Since distribution of GLP-1R in the kidney remains controversial, the renoprotective effects of GLP-1RAs may be partially explained by this mechanism.

### Glycemic Control

Although the glucose-independent mechanisms are emphasized, glycemic control by GLP-1RAs is considered to be involved in its renoprotective effects. In LEADER, the use of liraglutide was associated with a 0.4% HbA1c reduction compared with the placebo (Marso et al., 2016b; Zinman et al., 2018). As a reduction of 0.5% in HbA1c is a clinically important difference (Zinman et al., 2018), a greater reduction by liraglutide may contribute to the renoprotective effects. Furthermore, liraglutide use was associated with a reduction in weight of 2.3 kg. In SUSTAIN-6, semaglutide use *vs*. placebo was associated with respective reductions in HbA1c of −0.66% (0.5 mg) *vs*. −1.05% (1.0 mg) and body weight of −2.9 kg (0.5 mg) *vs*. −4.4 kg (1.0 mg) (Kaul, 2018). In REWIND, dulaglutide use reduced the HbA1c value by −0.61% and body weight by −1.46 kg compared with placebo (Gerstein et al., 2019b). In these trails, GLP-1RAs exerted renoprotection, irrespective of the baseline HbA1c (Kristensen et al., 2019).

## Limitations of Renoprotection by GLP-1RAs

A series of experimental studies revealed that GLP-1RAs can exert renoprotective effects independent of their glucose-lowering activities; however, clinical evidence at present is insufficient to support these observations. For instance, there are no established methods for assessing the reduction in oxidative stress and inflammation by GLP-1RAs. It is difficult to evaluate the extent to which glucose-independent mechanisms are involved in renoprotection by GLP-1RAs. In clinical settings, glucose-lowering, weight loss, natriuresis, and blood pressure reduction may account for the renoprotective effects of GLP-1RAs. As described above, GLP-1RAs attenuate albuminuria and marginally reduce eGFR decline. However, whether or not albuminuria is a clinically relevant renal outcome remains unclear. Long-term clinical trials will be needed to address this question. The additive effects of combination treatment of GLP-1RAs and SGLT2 inhibitors on DKD are uncertain. In DELIGHT, the combination of saxagliptin and dapagliflozin showed potentially additive but marginal albuminuria-lowering effects in T2D subjects (Pollock et al., 2019). A meta-analysis showed that GLP-1RA and SGLT2 inhibitor combination therapy was associated with a greater reduction in HbA1c (−0.74%), body weight (−1.61 kg), and systolic blood pressure (−3.32 mmHg) than SGLT2 inhibitor monotherapy (Castellana et al., 2019), suggesting that this combination may induce additive renoprotective effects. Further studies will be required to address this issue.

## Conclusion and Perspectives

GLP-1RAs are widely used in the treatment of T2D. The treatment of DKD has been largely dependent on the management of hyperglycemia and hypertension. Thus, novel therapeutic approaches that exert renoprotective effects independently of these factors have been awaited. A series of clinical trials and experimental studies support the beneficial effects of GLP-1RAs on DKD. Lessons from clinical trials demonstrate these effects are mainly driven by reductions in albuminuria. In contrast, the beneficial effects of SGLT2 inhibitors on albuminuria and eGFR decline in DKD were demonstrated by EMPA-REG OUTCOME (Wanner et al., 2016), CANagliflozin cardioVascular Assessment Study (CANVAS) (Perkovic et al., 2018), DECLARE-TIMI58 (Mosenzon et al., 2019b), and Canagliflozin and Renal Events in Diabetes with Established Nephropathy Clinical Evaluation (CREDENCE) (Perkovic et al., 2019). It remains unclear why these differences were observed. The effects of SGLT2s inhibitors on hemodynamics and glomerular hyperfiltration seem to be robust whereas those of GLP-1RAs have not been established. In addition, the different distribution of SGLT2 and GLP-1R may be involved. Further studies are required to clarify the differences in their effects on the kidney and how to use them appropriately in clinical practice. Nevertheless, GLP-1RAs are a promising therapeutic option for DKD.

## Author Contributions

DK and YT wrote and revised the manuscript.

## Conflict of Interest

DK has received research support from Sanofi, Tanabe Pharma, Terumo, Böehringer Ingelheim, Kyowa Kirin, Sumitomo Dainippon Pharma, Ono Pharmaceutical and Takeda Pharmaceutical as well as speaker honoraria from Novo Nordisk Pharma, Sanofi, and Takeda Pharmaceutical.

The remaining author declares that the research was conducted in the absence of any commercial or financial relationships that could be construed as a potential conflict of interest.

## References

[B1] Avgerinos I. Karagiannis T. Malandris K. Liakos A. Mainou M. Bekiari E. (2019). Glucagon-like peptide-1 receptor agonists and microvascular outcomes in type 2 diabetes: A systematic review and meta-analysis. Diabetes Obes. Metab. 21, 188–193. 10.1111/dom.13484 30058208

[B2] Bethel M. A. Mentz R. J. Merrill P. Buse J. B. Chan J. C. Goodman S. G. (2020). Microvascular and Cardiovascular Outcomes According to Renal Function in Patients Treated With Once-Weekly Exenatide: Insights From the EXSCEL Trial. Diabetes Care 43, 446–452. 10.2337/dc19-1065 31757838 PMC7411285

[B3] Carraro-Lacroix L. R. Malnic G. Girardi A. C. (2009). Regulation of Na+/H+ exchanger NHE3 by glucagon-like peptide 1 receptor agonist exendin-4 in renal proximal tubule cells. Am. J. Physiol. Renal Physiol. 297, F1647–F1655. 10.1152/ajprenal.00082.2009 19776173

[B4] Castellana M. Cignarelli A. Brescia F. Perrini S. Natalicchio A. Laviola L. (2019). Efficacy and safety of GLP-1 receptor agonists as add-on to SGLT2 inhibitors in type 2 diabetes mellitus: A meta-analysis. Sci. Rep. 9, 19351. 10.1038/s41598-019-55524-w 31852920 PMC6920368

[B5] Chang J. T. Liang Y. J. Hsu C. Y. Chen C. Y. Chen P. J. Yang Y. F. (2017). Glucagon-like peptide receptor agonists attenuate advanced glycation end products-induced inflammation in rat mesangial cells. BMC Pharmacol. Toxicol. 18, 67. 10.1186/s40360-017-0172-3 29065926 PMC5655807

[B6] Chen S. J. Lv L. L. Liu B. C. Tang R. N. (2020). Crosstalk between tubular epithelial cells and glomerular endothelial cells in diabetic kidney disease. Cell Prolif. 53, e12763. 10.1111/cpr.12763 31925859 PMC7106959

[B7] Crajoinas R. O. Oricchio F. T. Pessoa T. D. Pacheco B. P. Lessa L. M. Malnic G. (2011). Mechanisms mediating the diuretic and natriuretic actions of the incretin hormone glucagon-like peptide-1. Am. J. Physiol. Renal Physiol. 301, F355–F363. 10.1152/ajprenal.00729.2010 21593184

[B8] Cui R. Tian L. Lu D. Li H. Cui J. (2019). Exendin-4 Protects Human Retinal Pigment Epithelial Cells from H2O2-Induced Oxidative Damage via Activation of NRF2 Signaling. Ophthalmic Res., 10.1159/000504891 31865348

[B9] Davies M. J. Bergenstal R. Bode B. Kushner R. F. Lewin A. Skjoth T. V. (2015). Efficacy of Liraglutide for Weight Loss Among Patients With Type 2 Diabetes: The SCALE Diabetes Randomized Clinical Trial. JAMA 314, 687–699. 10.1001/jama.2015.9676 26284720

[B10] Davies M. J. Bain S. C. Atkin S. L. Rossing P. Scott D. Shamkhalova M. S. (2016). Efficacy and Safety of Liraglutide Versus Placebo as Add-on to Glucose-Lowering Therapy in Patients With Type 2 Diabetes and Moderate Renal Impairment (LIRA-RENAL): A Randomized Clinical Trial. Diabetes Care 39, 222–230. 26681713 10.2337/dc14-2883

[B11] De Lucas M. D. G. Bueno B. A. Sierra J. O. (2017). Liraglutide preserves renal function in overweight diabetic patients with stage 3 chronic kidney disease. Eur. J. Intern Med. 44, e28–e29. 10.1016/j.ejim.2017.07.020 28711390

[B12] Drucker D. J. (2018). The Ascending GLP-1 Road From Clinical Safety to Reduction of Cardiovascular Complications. Diabetes 67, 1710–1719. 10.2337/dbi18-0008 30135132

[B13] Farah L. X. Valentini V. Pessoa T. D. Malnic G. Mcdonough A. A. Girardi A. C. (2016). The physiological role of glucagon-like peptide-1 in the regulation of renal function. Am. J. Physiol. Renal Physiol. 310, F123–F127. 10.1152/ajprenal.00394.2015 26447224 PMC5504384

[B14] Gerstein H. C. Colhoun H. M. Dagenais G. R. Diaz R. Lakshmanan M. Pais P. (2019a). Dulaglutide and renal outcomes in type 2 diabetes: an exploratory analysis of the REWIND randomised, placebo-controlled trial. Lancet 394, 131–138. 10.1016/S0140-6736(19)31150-X 31189509

[B15] Gerstein H. C. Colhoun H. M. Dagenais G. R. Diaz R. Lakshmanan M. Pais P. (2019b). Dulaglutide and cardiovascular outcomes in type 2 diabetes (REWIND): a double-blind, randomised placebo-controlled trial. Lancet 394, 121–130. 10.1016/S0140-6736(19)31149-3 31189511

[B16] Giugliano D. Maiorino M. I. Bellastella G. Longo M. Chiodini P. Esposito K. (2019). GLP-1 receptor agonists for prevention of cardiorenal outcomes in type 2 diabetes: An updated meta-analysis including the REWIND and PIONEER 6 trials. Diabetes Obes. Metab. 21, 2576–2580. 10.1111/dom.13847 31373167

[B17] Gutzwiller J. P. Tschopp S. Bock A. Zehnder C. E. Huber A. R. Kreyenbuehl M. (2004). Glucagon-like peptide 1 induces natriuresis in healthy subjects and in insulin-resistant obese men. J. Clin. Endocrinol. Metab. 89, 3055–3061. 10.1210/jc.2003-031403 15181098

[B18] Han F. Hou N. Liu Y. Huang N. Pan R. Zhang X. (2019). Liraglutide improves vascular dysfunction by regulating a cAMP-independent PKA-AMPK pathway in perivascular adipose tissue in obese mice. BioMed. Pharmacother. 120, 109537. 10.1016/j.biopha.2019.109537 31605951

[B19] Helmstadter J. Frenis K. Filippou K. Grill A. Dib M. Kalinovic S. (2020). Endothelial GLP-1 (Glucagon-Like Peptide-1) Receptor Mediates Cardiovascular Protection by Liraglutide In Mice With Experimental Arterial Hypertension. Arterioscler. Thromb. Vasc. Biol. 40, 145–158. 10.1161/atv.0000615456.97862.30 31747801 PMC6946108

[B20] Hendarto H. Inoguchi T. Maeda Y. Ikeda N. Zheng J. Takei R. (2012). GLP-1 analog liraglutide protects against oxidative stress and albuminuria in streptozotocin-induced diabetic rats via protein kinase A-mediated inhibition of renal NAD(P)H oxidases. Metabolism 61, 1422–1434. 10.1016/j.metabol.2012.03.002 22554832

[B21] Hernandez A. F. Green J. B. Janmohamed S. D’agostino R.B. Granger C. B. Jones N. P. (2018). Albiglutide and cardiovascular outcomes in patients with type 2 diabetes and cardiovascular disease (Harmony Outcomes): a double-blind, randomised placebo-controlled trial. Lancet 392, 1519–1529. 10.1016/S0140-6736(18)32261-X 30291013

[B22] Holman R. R. Bethel M. A. Mentz R. J. Thompson V. P. Lokhnygina Y. Buse J. B. (2017). Effects of Once-Weekly Exenatide on Cardiovascular Outcomes in Type 2 Diabetes. N. Engl. J. Med. 377, 1228–1239. 10.1056/NEJMoa1612917 28910237 PMC9792409

[B23] Honda J. Kimura T. Sakai S. Maruyama H. Tajiri K. Murakoshi N. (2018). The glucagon-like peptide-1 receptor agonist liraglutide improves hypoxia-induced pulmonary hypertension in mice partly via normalization of reduced ET(B) receptor expression. Physiol. Res. 67, S175–S184. 10.33549/physiolres.933822 29947538

[B24] Husain M. Birkenfeld A. L. Donsmark M. Dungan K. Eliaschewitz F. G. Franco D. R. (2019). Oral Semaglutide and Cardiovascular Outcomes in Patients with Type 2 Diabetes. N. Engl. J. Med. 381, 841–851. 10.1056/NEJMoa1901118 31185157

[B25] Hviid A. V. R. Sorensen C. M. (2020). Glucagon-like peptide-1 receptors in the kidney: impact on renal autoregulation. Am. J. Physiol. Renal Physiol. 318, F443–F454. 10.1152/ajprenal.00280.2019 31841385

[B26] Ito M. Tanaka T. Nangaku M. (2020). Nuclear factor erythroid 2-related factor 2 as a treatment target of kidney diseases. Curr. Opin. Nephrol. Hypertens. 29, 128–135. 10.1097/MNH.0000000000000556 31592832

[B27] Jensen E. P. Poulsen S. S. Kissow H. Holstein-Rathlou N. H. Deacon C. F. Jensen B. L. (2015). Activation of GLP-1 receptors on vascular smooth muscle cells reduces the autoregulatory response in afferent arterioles and increases renal blood flow. Am. J. Physiol. Renal Physiol. 308, F867–F877. 10.1152/ajprenal.00527.2014 25656368

[B28] Jia Y. Zheng Z. Guan M. Zhang Q. Li Y. Wang L. (2018). Exendin-4 ameliorates high glucose-induced fibrosis by inhibiting the secretion of miR-192 from injured renal tubular epithelial cells. Exp. Mol. Med. 50, 1–13. 10.1038/s12276-018-0084-3 PMC5938044 29717107

[B29] Kang Z. Zeng J. Zhang T. Lin S. Gao J. Jiang C. (2019). Hyperglycemia induces NF-kappaB activation and MCP-1 expression via downregulating GLP-1R expression in rat mesangial cells: inhibition by metformin. Cell Biol. Int. 43, 940–953. 10.1002/cbin.11184 31136032

[B30] Kaul S. (2017). Erratum. Mitigating Cardiovascular Risk in Type 2 Diabetes With Antidiabetes Drugs: A Review of Principal Cardiovascular Outcome Results of EMPA-REG OUTCOME, LEADER, and SUSTAIN-6 Trials. Diabetes Care 40, 821–831. 10.2337/dc17-0291 28637887

[B31] Kawanami D. Matoba K. Utsunomiya K. (2016). Signaling pathways in diabetic nephropathy. Histol. Histopathol., 31 (10), 1059–1067 10.14670/HH-11-777 27094540

[B32] Kim M. Platt M. J. Shibasaki T. Quaggin S. E. Backx P. H. Seino S. (2013). GLP-1 receptor activation and Epac2 link atrial natriuretic peptide secretion to control of blood pressure. Nat. Med. 19, 567–575. 10.1038/nm.3128 23542788

[B33] Kodera R. Shikata K. Kataoka H. U. Takatsuka T. Miyamoto S. Sasaki M. (2011). Glucagon-like peptide-1 receptor agonist ameliorates renal injury through its anti-inflammatory action without lowering blood glucose level in a rat model of type 1 diabetes. Diabetologia 54, 965–978. 10.1007/s00125-010-2028-x 21253697

[B34] Kristensen S. L. Rorth R. Jhund P. S. Docherty K. F. Sattar N. Preiss D. (2019). Cardiovascular, mortality, and kidney outcomes with GLP-1 receptor agonists in patients with type 2 diabetes: a systematic review and meta-analysis of cardiovascular outcome trials. Lancet Diabetes Endocrinol. 7, 776–785. 10.1016/S2213-8587(19)30249-9 31422062

[B35] Lee J. W. Chou C. L. Knepper M. A. (2015). Deep Sequencing in Microdissected Renal Tubules Identifies Nephron Segment-Specific Transcriptomes. J. Am. Soc. Nephrol. 26, 2669–2677. 10.1681/ASN.2014111067 25817355 PMC4625681

[B36] Li W. Cui M. Wei Y. Kong X. Tang L. Xu D. (2012). Inhibition of the expression of TGF-beta1 and CTGF in human mesangial cells by exendin-4, a glucagon-like peptide-1 receptor agonist. Cell Physiol. Biochem. 30, 749–757. 10.1159/000341454 22890152

[B37] Li N. Zhao Y. Yue Y. Chen L. Yao Z. Niu W. (2016). Liraglutide ameliorates palmitate-induced endothelial dysfunction through activating AMPK and reversing leptin resistance. Biochem. Biophys. Res. Commun. 478, 46–52. 10.1016/j.bbrc.2016.07.095 27457805

[B38] Li Y. K. Ma D. X. Wang Z. M. Hu X. F. Li S. L. Tian H. Z. (2018). The glucagon-like peptide-1 (GLP-1) analog liraglutide attenuates renal fibrosis. Pharmacol. Res. 131, 102–111. 10.1016/j.phrs.2018.03.004 29530599

[B39] Liljedahl L. Pedersen M. H. Mcguire J. N. James P. (2019). The impact of the glucagon-like peptide 1 receptor agonist liraglutide on the streptozotocin-induced diabetic mouse kidney proteome. Physiol. Rep. 7, e13994. 10.14814/phy2.13994 30806030 PMC6389751

[B40] Mann J. F. E. Orsted D. D. Brown-Frandsen K. Marso S. P. Poulter N. R. Rasmussen S. (2017). Liraglutide and Renal Outcomes in Type 2 Diabetes. N. Engl. J. Med. 377, 839–848. 10.1056/NEJMoa1616011 28854085

[B41] Mann J. F. E. Fonseca V. Mosenzon O. Raz I. Goldman B. Idorn T. (2018). Effects of Liraglutide Versus Placebo on Cardiovascular Events in Patients With Type 2 Diabetes Mellitus and Chronic Kidney Disease. Circulation 138, 2908–2918. 10.1161/CIRCULATIONAHA.118.036418 30566006 PMC6296845

[B42] Marso S. P. Bain S. C. Consoli A. Eliaschewitz F. G. Jodar E. Leiter L. A. (2016a). Semaglutide and Cardiovascular Outcomes in Patients with Type 2 Diabetes. N. Engl. J. Med. 375, 1834–1844. 10.1056/NEJMoa1607141 27633186

[B43] Marso S. P. Daniels G. H. Brown-Frandsen K. Kristensen P. Mann J. F. Nauck M. A. (2016b). Liraglutide and Cardiovascular Outcomes in Type 2 Diabetes. N. Engl. J. Med. 375 (4), 311–322. 10.1056/NEJMoa1603827 27295427 PMC4985288

[B44] Moellmann J. Klinkhammer B. M. Onstein J. Stohr R. Jankowski V. Jankowski J. (2018). Glucagon-Like Peptide 1 and Its Cleavage Products Are Renoprotective in Murine Diabetic Nephropathy. Diabetes 67, 2410–2419. 10.2337/db17-1212 30104246

[B45] Mosenzon O. Blicher T. M. Rosenlund S. Eriksson J. W. Heller S. Hels O. H. (2019a). Efficacy and safety of oral semaglutide in patients with type 2 diabetes and moderate renal impairment (PIONEER 5): a placebo-controlled, randomised, phase 3a trial. Lancet Diabetes Endocrinol. 7, 515–527. 10.2337/db19-1004-P 31189517

[B46] Mosenzon O. Wiviott S. D. Cahn A. Rozenberg A. Yanuv I. Goodrich E. L. (2019b). Effects of dapagliflozin on development and progression of kidney disease in patients with type 2 diabetes: an analysis from the DECLARE-TIMI 58 randomised trial. Lancet Diabetes Endocrinol. 7, 606–617. 10.1016/S2213-8587(19)30180-9 31196815

[B47] Muskiet M. H. Tonneijck L. Smits M. M. Kramer M. H. Diamant M. Joles J. A. (2016). Acute renal haemodynamic effects of glucagon-like peptide-1 receptor agonist exenatide in healthy overweight men. Diabetes Obes. Metab. 18, 178–185. 10.1111/dom.12601 26636423

[B48] Muskiet M. H. A. Tonneijck L. Smits M. M. Van Baar M. J. B. Kramer M. H. H. Hoorn E. J. (2017). GLP-1 and the kidney: from physiology to pharmacology and outcomes in diabetes. Nat. Rev. Nephrol. 13, 605–628. 10.1038/nrneph.2017.123 28869249

[B49] Muskiet M. H. A. Tonneijck L. Huang Y. Liu M. Saremi A. Heerspink H. J. L. (2018). Lixisenatide and renal outcomes in patients with type 2 diabetes and acute coronary syndrome: an exploratory analysis of the ELIXA randomised, placebo-controlled trial. Lancet Diabetes Endocrinol. 6, 859–869. 10.1016/S2213-8587(18)30268-7 30292589

[B50] Muskiet M. H. A. Bunck M. C. Heine R. J. Corner A. Yki-Jarvinen H. Eliasson B. (2019). Exenatide twice-daily does not affect renal function or albuminuria compared to titrated insulin glargine in patients with type 2 diabetes mellitus: A post-hoc analysis of a 52-week randomised trial. Diabetes Res. Clin. Pract. 153, 14–22. 10.1016/j.diabres.2019.05.001 31078666

[B51] Perkovic V. De Zeeuw D. Mahaffey K. W. Fulcher G. Erondu N. Shaw W. (2018). Canagliflozin and renal outcomes in type 2 diabetes: results from the CANVAS Program randomised clinical trials. Lancet Diabetes Endocrinol. 6, 691–704. 10.1016/S2213-8587(18)30141-4 29937267

[B52] Perkovic V. Jardine M. J. Neal B. Bompoint S. Heerspink H. J. L. Charytan D. M. (2019). Canagliflozin and Renal Outcomes in Type 2 Diabetes and Nephropathy. N. Engl. J. Med. 380, 2295–2306. 10.1056/NEJMoa1811744 30990260

[B53] Pfeffer M. A. Claggett B. Diaz R. Dickstein K. Gerstein H. C. Kober L. V. (2015). Lixisenatide in Patients with Type 2 Diabetes and Acute Coronary Syndrome. N. Engl. J. Med. 373, 2247–2257. 10.1056/NEJMoa1509225 26630143

[B54] Pollock C. Stefansson B. Reyner D. Rossing P. Sjostrom C. D. Wheeler D. C. (2019). Albuminuria-lowering effect of dapagliflozin alone and in combination with saxagliptin and effect of dapagliflozin and saxagliptin on glycaemic control in patients with type 2 diabetes and chronic kidney disease (DELIGHT): a randomised, double-blind, placebo-controlled trial. Lancet Diabetes Endocrinol. 7, 429–441. 10.1016/S2213-8587(19)30086-5 30992195

[B55] Pyke C. Heller R. S. Kirk R. K. Orskov C. Reedtz-Runge S. Kaastrup P. (2014). GLP-1 receptor localization in monkey and human tissue: novel distribution revealed with extensively validated monoclonal antibody. Endocrinology 155, 1280–1290. 10.1210/en.2013-1934 24467746

[B56] Rawshani A. Rawshani A. Gudbjornsdottir S. (2018). Smoking and Other Risk Factors in Type 2 Diabetes. N. Engl. J. Med. 379, 2575. 10.1056/NEJMoa1800256 30586516

[B57] Rodriguez R. Escobedo B. Lee A. Y. Thorwald M. Godoy-Lugo J. A. Nakano D. (2020). Simultaneous angiotensin receptor blockade and glucagon-like peptide-1 receptor activation ameliorate albuminuria in obese insulin-resistant rats. Clin. Exp. Pharmacol. Physiol. 47, 422–431. 10.1111/1440-1681.13206 31675433

[B58] Ronn J. Jensen E. P. Wewer Albrechtsen N. J. Holst J. J. Sorensen C. M. (2017). Glucagon-like peptide-1 acutely affects renal blood flow and urinary flow rate in spontaneously hypertensive rats despite significantly reduced renal expression of GLP-1 receptors. Physiol. Rep. 5, e13503. 10.14814/phy2.13503 PMC5727271 29233907

[B59] Schlatter P. Beglinger C. Drewe J. Gutmann H. (2007). Glucagon-like peptide 1 receptor expression in primary porcine proximal tubular cells. Regul. Pept. 141, 120–128. 10.1016/j.regpep.2006.12.016 17276524

[B60] Schultheis P. J. Clarke L. L. Meneton P. Miller M. L. Soleimani M. Gawenis L. R. (1998). Renal and intestinal absorptive defects in mice lacking the NHE3 Na+/H+ exchanger. Nat. Genet. 19, 282–285. 10.1038/969 9662405

[B61] Skov J. Dejgaard A. Frokiaer J. Holst J. J. Jonassen T. Rittig S. (2013). Glucagon-like peptide-1 (GLP-1): effect on kidney hemodynamics and renin-angiotensin-aldosterone system in healthy men. J. Clin. Endocrinol. Metab. 98, E664–E671. 10.1210/jc.2012-3855 23463656

[B62] Sukumaran V. Tsuchimochi H. Sonobe T. Shirai M. Pearson J. T. (2019). Liraglutide Improves Renal Endothelial Function in Obese Zucker Rats on a High-Salt Diet. J. Pharmacol. Exp. Ther. 369, 375–388. 10.1124/jpet.118.254821 30910920

[B63] Tonneijck L. Muskiet M. H. A. Blijdorp C. J. Smits M. M. Twisk J. W. Kramer M. H. H. (2019). Renal tubular effects of prolonged therapy with the GLP-1 receptor agonist lixisenatide in patients with type 2 diabetes mellitus. Am. J. Physiol. Renal Physiol. 316, F231–F240. 10.1152/ajprenal.00432.2018 30353743

[B64] Tuttle K. R. Lakshmanan M. C. Rayner B. Busch R. S. Zimmermann A. G. Woodward D. B. (2018). Dulaglutide versus insulin glargine in patients with type 2 diabetes and moderate-to-severe chronic kidney disease (AWARD-7): a multicentre, open-label, randomised trial. Lancet Diabetes Endocrinol. 6, 605–617. 10.1016/S2213-8587(18)30104-9 29910024

[B65] Van Der Aart-Van Der Beek A. B. Van Raalte D. H. Guja C. Hoogenberg K. Suchower L. J. Hardy E. (2020). Exenatide once weekly decreases urinary albumin excretion in patients with type 2 diabetes and elevated albuminuria: pooled analysis of randomized active controlled clinical trials. Diabetes Obes. Metab. 10.1111/dom.14067 PMC7496075 32329160

[B66] Von Scholten B. J. Persson F. Rosenlund S. Hovind P. Faber J. Hansen T. W. (2017). The effect of liraglutide on renal function: A randomized clinical trial. Diabetes Obes. Metab. 19, 239–247. 10.1111/dom.12808 27753201

[B67] Wang C. Li C. Peng H. Ye Z. Zhang J. Liu X. (2014). Activation of the Nrf2-ARE pathway attenuates hyperglycemia-mediated injuries in mouse podocytes. Cell Physiol. Biochem. 34, 891–902. 10.1159/000366307 25200066

[B68] Wang C. Li L. Liu S. Liao G. Li L. Chen Y. (2018). GLP-1 receptor agonist ameliorates obesity-induced chronic kidney injury via restoring renal metabolism homeostasis. PloS One 13, e0193473. 10.1371/journal.pone.0193473 29590132 PMC5873987

[B69] Wang X. Li Z. Huang X. Li F. Liu J. Li Z. (2019). An experimental study of exenatide effects on renal injury in diabetic rats1. Acta Cir. Bras. 34, e20190010000001. 10.1590/s0102-865020190010000001 30785502 PMC6585921

[B70] Wanner C. Inzucchi S. E. Lachin J. M. Fitchett D. Von Eynatten M. Mattheus M. (2016). Empagliflozin and Progression of Kidney Disease in Type 2 Diabetes. N. Engl. J. Med. 375, 323–334. 10.1056/NEJMoa1515920 27299675

[B71] Xu W. W. Guan M. P. Zheng Z. J. Gao F. Zeng Y. M. Qin Y. (2014). Exendin-4 alleviates high glucose-induced rat mesangial cell dysfunction through the AMPK pathway. Cell Physiol. Biochem. 33, 423–432. 10.1159/000358623 24556697

[B72] Ye Y. Zhong X. Li N. Pan T. (2019). Protective effects of liraglutide on glomerular podocytes in obese mice by inhibiting the inflammatory factor TNF-alpha-mediated NF-kappaB and MAPK pathway. Obes. Res. Clin. Pract. 13, 385–390. 10.1016/j.orcp.2019.03.003 30952571

[B73] Yin Q. H. Zhang R. Li L. Wang Y. T. Liu J. P. Zhang J. (2016). Exendin-4 Ameliorates Lipotoxicity-induced Glomerular Endothelial Cell Injury by Improving ABC Transporter A1-mediated Cholesterol Efflux in Diabetic apoE Knockout Mice. J. Biol. Chem. 291, 26487–26501. 10.1074/jbc.M116.730564 27784780 PMC5159509

[B74] Yin W. Xu S. Wang Z. Liu H. Peng L. Fang Q. (2018). Recombinant human GLP-1(rhGLP-1) alleviating renal tubulointestitial injury in diabetic STZ-induced rats. Biochem. Biophys. Res. Commun. 495, 793–800. 10.1016/j.bbrc.2017.11.076 29137984

[B75] Yin W. Jiang Y. Xu S. Wang Z. Peng L. Fang Q. (2019). Protein kinase C and protein kinase A are involved in the protection of recombinant human glucagon-like peptide-1 on glomeruli and tubules in diabetic rats. J. Diabetes Invest. 10, 613–625. 10.1111/jdi.12956 PMC6497589 30307132

[B76] Zhao Q. Xu H. Zhang L. Liu L. Wang L. (2019). GLP-1 receptor agonist lixisenatide protects against high free fatty acids-induced oxidative stress and inflammatory response. Artif. Cells Nanomed. Biotechnol. 47, 2325–2332. 10.1080/21691401.2019.1620248 31174433

[B77] Zhou S. J. Bai L. Lv L. Chen R. Li C. J. Liu X. Y. (2014). Liraglutide ameliorates renal injury in streptozotocininduced diabetic rats by activating endothelial nitric oxide synthase activity via the downregulation of the nuclear factorkappaB pathway. Mol. Med. Rep. 10, 2587–2594. 10.3892/mmr.2014.2555 25215431

[B78] Zhou T. Zhang M. Zhao L. Li A. Qin X. (2016). Activation of Nrf2 contributes to the protective effect of Exendin-4 against angiotensin II-induced vascular smooth muscle cell senescence. Am. J. Physiol. Cell Physiol. 311, C572–C582. 10.1152/ajpcell.00093.2016 27488664

[B79] Zinman B. Nauck M. A. Bosch-Traberg H. Frimer-Larsen H. Orsted D. D. Buse J. B. (2018). Liraglutide and Glycaemic Outcomes in the LEADER Trial. Diabetes Ther. 9, 2383–2392. 10.1007/s13300-018-0524-z 30392095 PMC6250637

[B80] Zitman-Gal T. Einbinder Y. Ohana M. Katzav A. Kartawy A. Benchetrit S. (2019). Effect of liraglutide on the Janus kinase/signal transducer and transcription activator (JAK/STAT) pathway in diabetic kidney disease in db/db mice and in cultured endothelial cells. J. Diabetes 11, 656–664. 10.1111/1753-0407.12891 30575282

